# Supplementary material for: Structural insight into TPX2-stimulated microtubule assembly
Source: eLife. 2017 Nov 9;6:e30959. doi: 10.7554/eLife.30959 (PMC5679754; doi:10.7554/eLife.30959)
Supplement: Figure 5—figure supplement 1—source data 1. [file elife-30959-fig5-figsupp1-data1.docx]

**Figure 5—source data 1. Lattice Parameters for Different MT States.**

|  | **Dimer rise (Å)** | **Dimer twist (°)** | **3-start rise (Å)** | **3-start twist (°)** |
| --- | --- | --- | --- | --- |
| GMPCPP-TPX2^mini^ 13-PF | 84.24 | 0.34 | 9.73 | -27.65 |
| GMPCPP-TPX2^mini^ 14-PF | 84.18 | -0.23 | 9.03 | -25.74 |
| GMPCPP-TPX2^micro^ 13-PF | 84.27 | 0.35 | 9.74 | -27.65 |
| GMPCPP-TPX2^micro^ 14-PF | 84.23 | -0.23 | 9.04 | -25.74 |
| GMPCPP (apo) 13-PF | 83.70 | 0.21 | 9.67 | -27.67 |
| GMPCPP (apo) 14-PF | 83.87 | -0.34 | 9.00 | -25.75 |
| GMPCPP-Kinesin 13-PF | 83.16 | 0.20 | 9.62 | -27.67 |
| GMPCPP-Kinesin 14-PF | 83.10 | -0.34 | 8.92 | -25.75 |

The helical parameters (rise and twist) related to the 3-start helix of the tubulin monomer and 13- or 14-start helix of the tubulin dimer are measured directly from the C1 reconstruction of each MT state.
